# Influence of content and intensity of thought on behavioral and pupil changes during active mind-wandering, off-focus, and on-task states

**DOI:** 10.3758/s13414-019-01865-7

**Published:** 2019-09-12

**Authors:** Esperanza Jubera-García, Wim Gevers, Filip Van Opstal

**Affiliations:** 1 Université Libre de Bruxelles, Brussels, Belgium; 2 University of Amsterdam, Amsterdam, The Netherlands

**Keywords:** Attention, Mind wandering, Off-focus state, SART, Pupil size

## Abstract

Mind wandering (MW) is a pervasive phenomenon that occurs very frequently, regardless of the task. A content-based definition of MW holds that it occurs when the content of thought switches from an ongoing task and/or an external stimulus-driven event to self-generated or inner thoughts. A recent account suggests that the transition between these different states of attention occurs via an off-focus state. Following this suggestion, previous work relating MW to pupil size might have lumped attentional states that are critically different from each (i.e., off-focus and MW states). In the present study, both behavior and pupil size were measured during a sustained-attention-to-response task, to disentangle the content of thought (on task or MW) from an off-focus state of mind. The off-focus state was operationalized by probing the intensity with which participants were on task or mind-wandering. The results of two experiments showed that the behavioral and phasic pupillary responses were sensitive to changes related to the content of thought. The behavioral responses were furthermore related to the intensity of the thought. However, no clear relation between the different attentional states and tonic pupillary diameter was found, suggesting that it is an unreliable proxy for MW.

According to recent research, we spend about 20% to 50% of our time awake with our thoughts away from what we are currently doing (Killingsworth & Gilbert, 2010; Seli et al., 2018). This phenomenon, generally labeled *mind wandering* (MW), is usually investigated with experience sampling that requires participants to report on their subjective ongoing experience while completing a task. An often-used experience-sampling method is the probe-caught method (Smallwood & Schooler, 2006), in which a probe is presented at random points in time that asks participants about their experience just prior to the probe. An example of such a thought probe is the following, “Prior to this question, were your thoughts on the task or on something else?,” which examines the metacognitive process of attention and has already revealed many interesting insights into MW (for reviews, see Christoff, Irving, Fox, Spreng, & Andrews-Hanna, 2016; Smallwood & Schooler, 2015).

This example thought probe fits with the idea that MW occurs when the content of thought switches from an ongoing task and/or an external stimulus-driven event to self-generated inner thoughts (Smallwood & Schooler, 2015). According to this content-based definition, MW is seen as having task-unrelated and/or stimulus-independent thoughts. However, as has been argued by recent reviews on the topic, this definition might not reflect important aspects of MW. Christoff et al. (2016) pointed out that the content-based definition of MW misses one of xzthe inherent characteristics of “wandering,” as defined by the Oxford English dictionary (Simpson, 1989): “to move hither and thither without fixed course or certain aim.” This resonates with other suggestions to separate intentional and unintentional MW episodes (Seli, Risko, Smilek, & Schacter, 2016), to distinguish between MW with awareness (“tune out”) versus without awareness (“zone out”) (Smallwood, McSpadden, & Schooler, 2007), or to differentiate between an exploratory off-focus state and an active MW state (Mittner, Hawkins, Boekel, & Forstmann, 2016). In fact, extending the concept of MW beyond the classical content-based division of thoughts might help clarify some outstanding issues in MW research.

One of these issues regards the relation between MW and pupil size. On the one hand, a clear relation between MW and the phasic pupil response has been provided in previous studies: When participants’ thoughts are not on the task, the response of the pupil to a visual stimulus is attenuated, as compared to when a participant is directing attention to the task (Kang, Huffer, & Wheatley, 2014; Mittner et al., 2014; Smallwood et al., 2011; Unsworth & Robison, 2016, 2018). On the other hand, however, the relation between MW and the tonic pupillary diameter (i.e., the baseline pupil diameter) is less clear. Some studies have reported the tonic diameter of the pupil to be smaller during MW than during on-task states (e.g., Grandchamp, Braboszcz, & Delorme, 2014; Huijser, van Vugt, & Taatgen, 2018; Mittner et al., 2014). Others have reported the exact opposite—namely, a larger tonic pupillary diameter during MW than during on-task states (e.g., Franklin, Broadway, Mrazek, Smallwood, & Schooler, 2013; Smallwood et al., 2012; Smallwood et al., 2011). These opposing results for the tonic diameter of the pupil might be—partially—attributable to differences in experimental designs between these studies (e.g., task demands, experience-sampling methods, etc.), but they might also be related to what these studies have in common, namely a content-based definition of MW.

A recent model of MW has suggested that the tonic diameter of the pupil is not necessarily different for different contents of thought (Mittner et al., 2016). In their model, Mittner and colleagues proposed three different attentional states: an on-task state, an active MW state, and an “off-focus” state. The on-task state and the active MW state are different from each other, in the sense that attention is focused toward a stimulus in the on-task state, and toward an internal event in the active MW state. However, these states are highly similar in terms of attentional focus: Both states require a strong focus on a specific thought, and are therefore termed *exploitative modes*. According to the model, the transition from an on-task state to an active MW state (or vice versa) occurs through an “off-focus” state that is very different from the other attentional states. In contrast to the on-task and active MW states, in the “off-focus” state there is no clear focus on a specific thought, and the attentional focus is broadened to allow the selection of a new thought; this attentional state is therefore more like an explorative mode. According to Mittner and colleagues, the locus coeruleus–norepinephrine (LC-NE) system is the driving force behind these attentional states, and because of the close relation between the LC-NE system and pupil size (Joshi, Li, Kalwani, & Gold, 2016), the different attentional states are all clearly linked to a certain tonic pupillary diameter. More specifically, the model predicts that the tonic pupillary diameter for both an active MW state and an on-task state should be the same, because both attentional states require the same attentional focus or intensity (i.e., they are both exploitative modes) and thus have the same, optimal tonic level of LC-NE activity. In contrast, an off-focus state would be more of an explorative mode, with higher arousal levels related to high tonic levels of the LC-NE system, and hence a larger pupil size. The tonic pupillary diameter thus critically depends on the intensity rather than the content of the thoughts. According to this suggestion, previous work relating pupil size to a content-based definition of MW might have lumped attentional states that are critically different from each other in terms of their relation with pupil size and subjective experience (i.e., an off-focus and an active MW state).

The main aim of the present study was thus to clarify the relation between the tonic pupillary diameter and MW, by testing the different states of attention as suggested by Mittner et al. (2016). This was done by adding an intensity dimension to the content-based thought probe. More specifically, while participants performed a sustained-attention-to-response task (SART; Robertson, Manly, Andrade, Baddeley, & Yiend, 1997), occasional thought probes were introduced that probed not only the *content* of thought (task related vs. task unrelated) but also the *intensity* of this thought (e.g., high vs. low intensity). Thoughts with low intensity were described to the participants as thoughts with no clear focus; thoughts with high intensity were described as thoughts with a clear focus that could be either task-related or task-unrelated (see the Appendix for the exact instructions). Reports of task-related and task-unrelated thoughts with high intensity would correspond to on-task and active MW episodes, respectively. Alternatively, reports of low intensity would be classified as an off-focus state of mind, regardless of whether the thoughts were task-related or task-unrelated. According to the model by Mittner et al. (2016), the tonic pupillary diameters should be similar for high-intensity states (active MW or on task), and higher for low-intensity (off-focus) states.

Interestingly, the model of Mittner et al. (2016) also relates differences in behavior to the different attentional states. Previous work has already shown that behavioral variability, as measured by the reaction time coefficient of variability (RTCV), can act as a reliable index of attentional disengagement (Allan Cheyne, Solman, Carriere, & Smilek, 2009). According to the model, behavioral variability should be smallest when participants are focused on the task, intermediate when their thoughts have no clear focus, and highest when participants are in active MW. If our intensity probe matches the notion of focus or intensity proposed by Mittner et al., then we would expect RTCV to be smallest for task-related reports with high intensity, intermediate for reports with low intensity (irrespective of whether they were task-related or task-unrelated), and highest for task-unrelated thoughts with high intensity.

This study consisted of two identical experiments. The sample size of the first experiment was calculated on the basis of the central limit theorem. To test the reliability of the result, a direct replication was performed (Exp. 2), with a sample size calculated from an a priori power analysis based on the results of the first experiment.

## Experiment 1

### Method

#### Participants

In our first experiment, 35 participants (mean age = 21.5 years, *SD* = 4.37; 25 females, 10 males) with normal vision completed the experiment for course credit, after giving their written informed consent in accordance with the Declaration of Helsinki. Participants were recruited from the student website of the Faculty of Psychology at the Université Libre de Bruxelles (ULB). The data from six participants were removed from further analyses due to unexpected building activities outside the lab during the experiment.

#### Stimuli and material

Testing was performed in a darkened room on a Mac OS X computer (version 10.7) and a screen (Samsung SMB 1940, 60-Hz refresh rate, 1,280 × 1,024 resolution). Participants positioned their heads on a chin rest at a distance of 60 cm from the top of the screen. Pupil diameter and gaze position for the dominant eye (Porac & Coren, 1976) were recorded with an EyeLink 1000 system (SR Research, Ottawa, Canada) at 500 Hz and were calibrated with a nine-point calibration prior to the experimental task. Stimulus presentation was programmed in Matlab (Mathworks, MA, USA) with the PsychToolbox extension (Brainard, 1997; Pelli, 1997).

#### Design and procedure

**Fig. 1:**
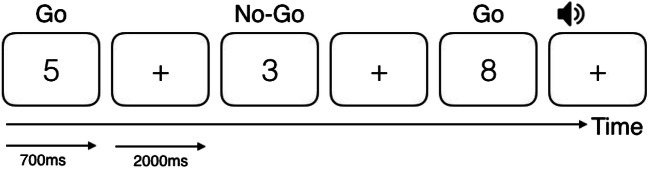
Sustained-attention-to-response task (SART) timeline. Participants had to respond to every number except to the number 3. An auditory cue introduced thought probes in a pseudorandom fashion every 30 to 50 s

The experiment was a modified version of the SART (Robertson et al., 1997). Because previous work had related the pace of the task to the appearance of MW episodes (Christoff, Gordon, Smallwood, Smith, & Schooler, 2009; Smallwood et al., 2004; Vinski & Watter, 2013), we slowed the pace of the task by increasing both the presentation time of the digits and the interstimulus interval (ISI), as compared to the original SART. Digits from 1 to 9 were presented at the center of the screen for 700 ms, interspersed with a fixation cross presented for 2,000 ms (Fig. 1). All stimuli were presented in black on a white background. Participants were instructed to press the space bar as fast as possible as soon as they saw a number appearing on the screen (go trials), but to withhold pressing when the number 3 appeared (no-go trials). No-go trials appeared in 11% of trials. The total number of trials was 783.

At pseudorandom points in time, an auditory signal probed participants to report their thoughts. The time between thought probes ranged from 30 to 50 s. The mean number of thought probes per participant was 50. On average, 18 of these 50 thought probes were preceded by a no-go trial. This allowed us to investigate the relationship between the no-go error rate and the attentional state. When a thought probe was presented, participants responded to two questions. The first question concerned the content of their thoughts prior to the presentation of the thought probe: Were the thoughts related to the task or not (i.e., on vs. off task)? The second question concerned the intensity of the focus of their thoughts prior to the thought probe. Intensity ranged from 1 (*low intensity*) to 4 (*high intensity*). Participants responded by pressing the keys “e” and “r,” for on and off task, respectively, and the keys {“u,” “i,” “o,” “p”} to report intensity, on an AZERTY keyboard. The keys were covered with stickers that stated “ON”–“OFF,” for the former, and that were numbered from 1 to 4, for the latter. For consistency, we will use the terms *content* and *intensity* of thought throughout the article to identify both variables. The Appendix provides a transcription of the instructions given to the participants at the start of the experiment. To familiarize participants with the task, they received training prior to the actual experiment that consisted of a shorter version of the experiment that finished after three thought probes had been presented. The duration of the experiment was 1 h.

#### Behavioral data analysis

Unless mentioned otherwise, statistical analyses were performed with linear mixed-effect models (LMMs) containing a random intercept across participants, to control for the dependency caused by the repeated sampling of data within participants (Pinheiro & Bates, 2000) and for the heterogeneity on the number of samples in each category of thought probe within and between participants. All analyses were run in R (version 3.3.2) using the lme4 package (Bates, Mächler, Bolker, & Walker, 2015). Stepwise model selection (Gelman & Hill, 2007) was based on the Akaike information criteria. The starting models were defined as the full models, containing all predictors and their respective two-way interaction. The predictors were content of thought (task related, task unrelated) and intensity (with four levels, from 1 to 4) as fixed factors. All *p* values were obtained by likelihood ratio tests of the full model against the model without the predictors’ interaction.

As in previous work (Bastian & Sackur, 2013), and to avoid possible disturbances in performance and/or attention caused by the prior thought probes, analyses were restricted to the eight trials (i.e., 21.6 s) before the thought probe. To study the influence of MW on participants’ performance, the reaction time coefficient of variability (RTCV) was investigated (Allan Cheyne et al., 2009; Seli, Cheyne, & Smilek, 2012; Vinski & Watter, 2013). The RTCV was calculated by taking the standard deviation of the eight trials before a thought probe, divided by their mean. No-go errors were not analyzed in relation to the thought probes, due to their small number of occurrences in every category, and were excluded from further analyses (percentage of no-go trials before a thought probe = 11%, percentage of no-go error trials before a thought probe = 3.36%).

#### Pupil data analysis

Prior to the analysis, blinks were linearly interpolated from 0.1 s before until 0.1 s after a blink (Kloosterman et al., 2015), and a low-pass, third-order Butterworth filter with a cutoff of 5 Hz was applied in order to remove high-frequency noise. Unless mentioned otherwise, the data for eight trials before a thought probe were selected, as in the behavioral data analysis. These data segments were then transformed to *z* scores per participant, to make the data comparable between subjects. Tonic pupillary diameter was investigated by removing the pupil’s phasic response associated with stimulus presentation or decisional and motor processes (Kang et al., 2014). This means that the tonic diameter was calculated as the mean pupil size between 500 ms before stimulus presentation and stimulus presentation for the eight trials. We also investigated the phasic response of the pupil, which was calculated by averaging the eight trials and subtracting the mean baseline value of the pupil (i.e., – 500 ms to stimulus presentation) from the maximum response of the pupil to the stimulus. The average values of the tonic and phasic pupillary response were used as dependent measures in the LMM analysis. The same LMMs were used as for the behavioral data analysis.

On average, the interpolated data due to blinks made up 5.21% of the data per participant. The stability of eye fixations was analyzed by mean centering their location per participant. Four participants had relatively unstable eye fixations (> 10% of their fixation data were more than 2 deg away from the center). Because this can distort a reliable measure of pupil size, these participants were removed from further analyses. The pupil analysis was thus performed on 25 participants. Inclusion of the four excluded participants, however, did not change the results.

### Results

#### Behavioral results

##### SART performance

In this first analysis, we investigated whether we could replicate previous results found with the SART. Because previous work had shown that RTs are faster before a failed than before a successful no-go trial, the mean RTs for the four go trials preceding either successful or failed no-go trials were calculated (similar to, e.g., Cheyne, Carriere, & Smilek, 2006; Robertson et al., 1997). In line with the previous work, a paired-samples *t* test revealed a significant difference between these two means, *t*(28) = 4.84, *p* < .001, indicating faster RTs before failed no-go trials (mean = 579 ms, *SD* = 79.88) than before successful no-go trials (mean = 606 ms, *SD* = 87.55).

##### Effects of attentional state on RTCV

**Fig. 2:**
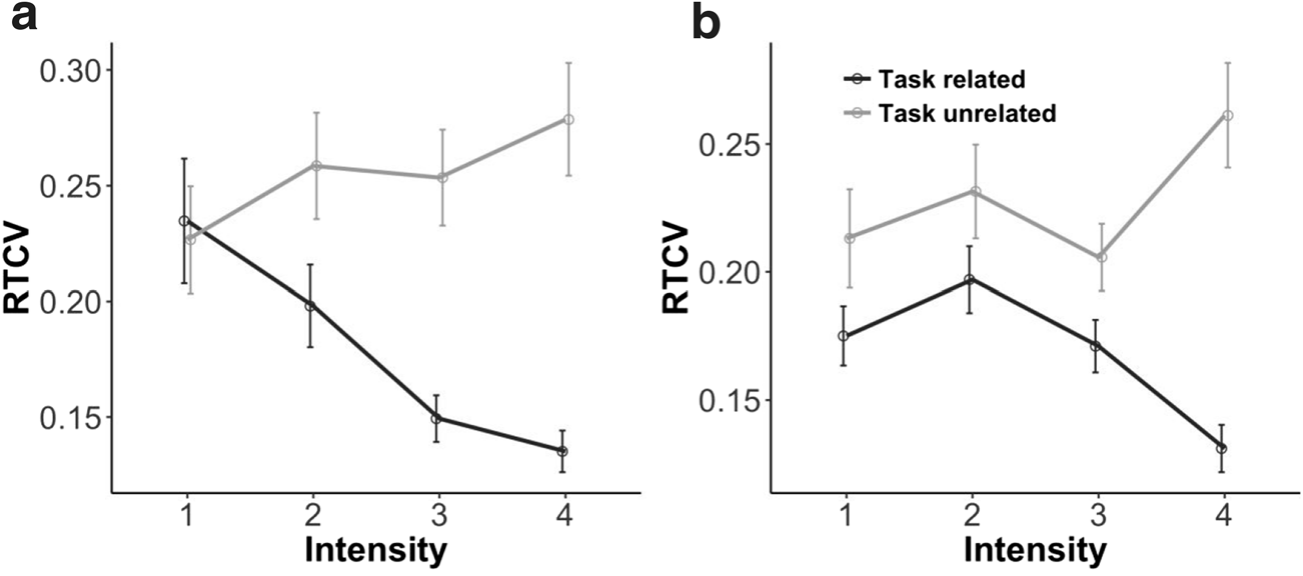
Interaction effect between content and intensity of thought on reaction time coefficients of variability (RTCVs) for (**a**) Experiment 1 and (**b**) Experiment 2. Error bars reflect the standard errors of the means

Because the model by Mittner et al. (2016) predicts that behavioral variability will be lowest for an on-task state, highest for an active MW state, and intermediate for an “off-focus” state, we tested the effects of the content and intensity of thought and their interaction on RTCV. This showed a significant effect of content of thought, *χ*^2^(1) = 13.68, *p* < .001, and a significant interaction effect, *χ*^2^(3) = 11.48, *p* = .009, but no effect of intensity, *χ*^2^(3) = 1.90, *p* = .59 (see Fig. 2a). Planned comparisons showed that RTCVs significantly decreased with increasing intensity for task-related thoughts, *χ*^2^(3) = 12.15, *p* = .007. For task-unrelated thoughts, the RTCV numerically, but not significantly, increased with increasing intensity, *χ*^2^(3) = 6.01, *p* = .11. No significant difference was observed between task-related and task-unrelated thoughts for the lowest intensity, *χ*^2^(1) = 0.40, *p* = .53.

#### Pupil results

##### SART and pupillary responses

**Fig. 3:**
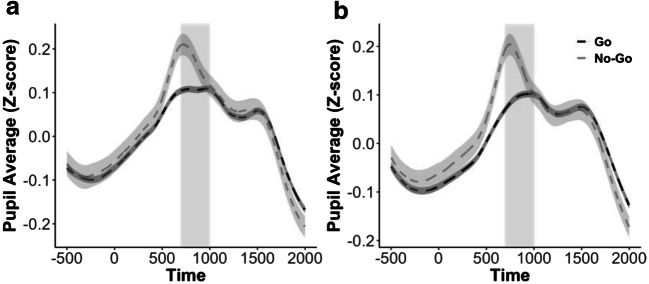
Phasic responses of the pupil were greater for no-go than for go trials in both (**a**) Experiment 1 and (**b**) Experiment 2. Shaded regions around the curves indicate the standard errors of the means. The areas with gray background denote the window of interest to test the effect of trial type on task-related pupil changes (i.e., 700 to 1,000 ms after stimulus onset)

As with the behavioral data, we first explored whether the pupil data would replicate the typical results observed in a SART. We therefore tested the effect of trial type (i.e., go vs. no-go trial) on the phasic response of the pupil. An LMM analysis on the difference in pupil size between 700 to 1,000 ms after stimulus onset showed a significant effect of trial type, *χ*^2^(1) = 5.16, *p* = .02. In line with previous work (Richer, Silverman, & Beatty, 1983; for a review, see Beatty & Lucero-Wagoner, 2000), the response of the pupil to the stimulus was larger in no-go trials than in go trials (Fig. 3a).

##### Effects of attentional state on tonic pupillary diameter

To investigate the effect of attentional state on mean pupil size, a full model with content and intensity of thought and their interaction was constructed. This revealed a significant effect of content of thought, *χ*^2^(1) = 6.11, *p* = .01, with a larger pupil size for task-unrelated thoughts. No effect of intensity, *χ*^2^(3) = 1.75, *p* = .63, or interaction between content and intensity, *χ*^2^(3) = 3.55, *p* = .31, was observed.

##### Effects of attentional state on phasic pupillary response

**Fig. 4:**
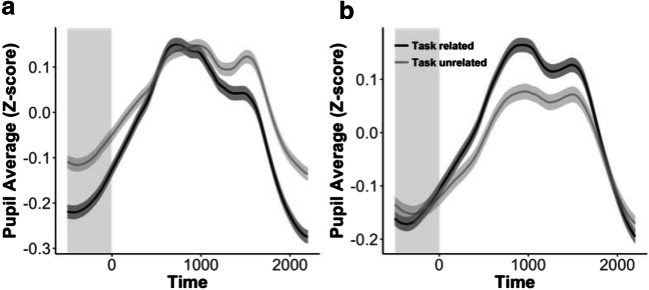
**a** Results from Experiment 1 showed that pupil size was related to the content of thought, with a larger tonic pupillary diameter when participants reported having task-unrelated (light-gray line) as compared to task-related (dark-gray line) thoughts. **b** This effect was not replicated in Experiment 2. Shaded regions around the curves indicate one standard error of the mean. The areas with gray background denote the window of interest to calculate the tonic pupillary diameter—that is, the baseline response (see Fig. 1)

The same analysis performed on the phasic response revealed a significant effect of the content of thought, *χ*^2^(1) = 7.42, *p* = .006, with a larger phasic response for task-related thoughts. No effect of intensity, *χ*^2^(3) = 3.72, *p* = .29, was observed (see Fig. 4a). An interaction between content and intensity, *χ*^2^(3) = 8.03, *p* = .045, showed that the difference in the phasic response between task-related and task-unrelated thoughts increased with increasing intensity. Post-hoc comparisons showed that the phasic response increased with increasing intensity for task-related thoughts, *χ*^2^(3) = 9.06, *p* = .03. For task-unrelated thoughts, no significant change was observed, *χ*^2^(3) = 0.60, *p* = .90.

### Discussion

This experiment aimed at clarifying the current controversies about the relation between MW and changes in pupil size. We therefore considered an on-task, an active MW, and an off-focus attentional state (Mittner et al., 2016) by adding an *intensity* probe to the commonly used *content* probe: Low-intensity thoughts (irrespective of the content of the thought) reflected an off-focus state, and high-intensity thoughts reflected either a focused on-task or an active MW state, depending on the content of the thought.

The results of this experiment replicated the typical results observed in a SART. The behavioral results showed that RTs were faster on trials prior to an error no-go trial than on trials prior to a successful no-go trial (Cheyne et al., 2006; Allan Cheyne et al., 2009; Robertson et al., 1997). Furthermore, the results from the pupil data showed a higher phasic pupillary response to the stimuli in no-go than in go trials (e.g., Richer et al., 1983).

The results furthermore revealed that the attentional state affected behavior: The RTCV was smallest when participants were highly focused on the task (i.e., having high-intensity task-related thoughts), and largest when participants were actively mind-wandering (i.e., having high-intensity task-unrelated thoughts). Interestingly, in line with the predictions from the model by Mittner et al. (2016), when participants reported being “off focus” (i.e., thoughts with low intensity), no difference in RTCVs was observed for task-related and task-unrelated thoughts, and the RTCV fell in-between the RTCVs of high-intensity task-related and task-unrelated states. This not only replicated previous findings showing that the RTCV is higher for an off-task than for an on-task state (Bastian & Sackur, 2013), but also suggests that our intensity probes adequately captured the on- and off-focus states proposed by Mittner et al. (2016).

The results of the pupil analyses also replicated earlier findings. The phasic response of the pupil to the stimulus presentation was increased during task-related as compared to task-unrelated thoughts (Mittner et al., 2014; Smallwood et al., 2011; Unsworth & Robison, 2016). In addition, this effect increased with increasing intensity. Most importantly, however, in contrast to the RTCV results, the effect of intensity could not be confirmed in the results for tonic pupillary diameter. Here, only a main effect of the content of thought was found, with a larger pupil size for task-unrelated than for task-related thoughts. Although this result fits with previous work showing an effect of the content of thought on the baseline pupil size (e.g., Franklin et al., 2013; Smallwood et al., 2012; Smallwood et al., 2011), it fails to support the suggestion that the intensity rather than the content of the thought is more predictive of pupil size.

Because the results from the tonic pupillary diameter in Experiment 1 ran counter to the predictions, and because the motivation for the sample size of Experiment 1 has been questioned (Cohen, 1990), the reliability of the most important results obtained in Experiment 1 was investigated in a second experiment that served as an exact replication. However, in contrast to Experiment 1, the sample size for the replication experiment was defined a priori on the basis of the key results of Experiment 1. We therefore calculated the observed power in the first experiment, to estimate the number of participants necessary to achieve high power (80%) in the LMM of the second experiment, with the R package SIMR (Green & Macleod, 2016). The power of the two key results in Experiment 1 was calculated. The first key result was the interaction effect of content and intensity of thought on RTCV. The power of this interaction resulted in a power of 84.70% by running a Kenward–Roger test on 1,000 simulations (Luke, 2017). The second key result was the effect of content of thought on the tonic pupillary diameter. A Kenward–Roger test on 1,000 simulations revealed a power of 86.30% for this result. Because a power of 80% is generally considered sufficient, the second experiment was performed with the same number of participants as in the first (i.e., 29 participants). Anticipating the possibility for minor data loss, we decided to test five additional participants (i.e., 34 in total).

## Experiment 2

### Method

#### Participants

A total of 34 participants (mean age = 20.8 years, *SD* = 3.71; 28 females, six males) with normal vision completed the experiment for course credit after giving their written informed consent in accordance with the Declaration of Helsinki. Participants were recruited via the student website of the Faculty of Psychology at the ULB. The data from one participant was removed because she fell asleep during the experiment (resulting in excessive high RTs and missing pupil data).

#### Materials and procedure

These were identical to those aspects of Experiment 1.

#### Behavioral data analysis

This was identical to that in Experiment 1. As in Experiment 1, the no-go errors were again not analyzed in relation to the thought probes, due to their small number of occurrences in every category, and were excluded from further analyses (percentage of no-go trials before a thought probe = 11%, percentage of no-go error trials before a thought probe = 3.02%).

#### Pupil data analysis

This was also as in Experiment 1. On average, the interpolated data due to blinks made up 5.88% of the analyzed data per participant. Five participants had unstable eye fixations (i.e., fixation was more than 2 deg away from the center for more than 10% of the data) and were therefore removed from further analyses. Inclusion of these participants in the analysis, however, did not change the results.

### Results

#### Behavioral results

##### SART performance

In line with the previous studies and with Experiment 1, a paired-samples *t* test revealed faster mean RTs before failed no-go trials (mean = 585 ms, *SD* = 68.15) than before successful no-go trials (mean = 604 ms, *SD* = 72.53), *t*(32) = 3.06, *p* = .004.

##### Effects of attentional state on RTCV

The analysis to test the effects of content and intensity of thought and their interaction on RTCVs replicated the findings of Experiment 1: a significant effect of content of thought, *χ*^2^(1) = 17.61, *p* < .001; a significant interaction effect, *χ*^2^(3) = 8.45, *p* = .038 (see Fig. 2b); and no effect of intensity, *χ*^2^(3) = 3.99, *p* = .26. Planned comparisons showed that RTCVs decreased with increasing intensity for task-related thoughts, *χ*^2^(3) = 8.85, *p* = .03. For task-unrelated thoughts, no significant increase in RTCVs was observed with increasing intensity: *χ*^2^(3) = 4.02, *p* = .26. No difference was found between task-related and task-unrelated thoughts at the lowest intensity, *χ*^2^(1) = 1.33, *p* = .25, as predicted. The interaction is presented in Fig. 2b. These results are highly similar to those in Experiment 1, with a clear decrease in RTCV for task-related thoughts with increasing intensity. Because the results were less clear for the task-unrelated thoughts—a numerical but nonsignificant increase in both experiments—the data of both experiments were combined, and the same analysis was performed. This now also revealed a significant effect of intensity for task-unrelated thoughts, *χ*^2^(3) = 8.48, *p* = .037.

#### Pupil results

##### SART and pupillary responses

Trial onset evoked an increase in pupil size that reached a peak around 800 ms during no-go trials, and a softer peak around 900 ms after stimulus presentation during go trials. This was confirmed by a model that tested the effect of trial type (i.e., go vs. no-go trial) on task-related pupil changes between 700 and 1,000 ms after stimulus onset. This revealed a significant effect of type of trial, *χ*^2^(1) = 8.39, *p* = .004, similar to the results of Experiment 1 (see Fig. 3b).

##### Effects of attentional state on tonic pupillary diameter

To investigate the effect of attentional state on pupil size, a full model with content and intensity of thought and their interaction was constructed. No effect of content of thought was found, *χ*^2^(1) = 0.19, *p* = *.*66 (different from in Exp. 1), nor an effect of intensity, *χ*^2^(3) = 0.25, *p* = .97, or an interaction, *χ*^2^(3) = 0.23, *p* = .97 (Fig. 4b).

##### Effects of attentional state on phasic pupillary response

A significant effect of content of thought was found, *χ*^2^(1) = 23.66, *p* < .001, with a larger poststimulus-from-baseline pupil difference being associated with task-related thoughts. No effect of intensity, *χ*^2^(3) = 5.71, *p* = .13, or interaction, *χ*^2^(3) = 5.08, *p* = .17, was observed.

### Discussion

The aim of this experiment was to test the reliability of the results obtained in Experiment 1. We therefore replicated the experiment with the same method and analyses and with a sample size derived from a power analysis on the two key results of Experiment 1.

All behavioral results were replicated. Most importantly, the effects of content and intensity of thought on the RTCV were identical to those observed in Experiment 1: High-intensity task-related and task-unrelated thoughts were related to the lowest and highest measures of RTCV, respectively. Off-focus thoughts, or thoughts with low intensity, had an intermediate RTCV, irrespective of the content of the thought.

A different pattern of results emerged from the pupil analyses. Similar to Experiment 1, the phasic pupillary response was again found to be reduced for task-unrelated as compared to task-related thoughts. However, the tonic pupillary diameter results, which had been different depending on the content of the thought in Experiment 1, did not replicate in this experiment.

## General discussion

This study aimed at disentangling the controversy on the relation between MW and tonic pupillary diameter. To do so, we followed the proposal of a recent model that relates differences in the tonic pupillary diameter to differences in the intensity of the thought (focused vs. not focused), rather than to differences in the content of the thought (task related vs. unrelated) (Mittner et al., 2016). According to this model, the tonic pupillary diameters should be similar for focused thoughts, irrespective of the content of the thought (i.e., similar responses for an on-task state and an active MW state), but larger for “off-focus” thoughts with no clear content. The model also predicts that behavioral variability should be smallest when participants are having on-task thoughts, intermediate when they have “off-focus” thoughts, and highest when they are actively mind-wandering.

The most important outcome of the present study is that the behavioral and phasic pupil results replicated, whereas the tonic pupil results were inconsistent. Indeed, the results of the tonic pupillary diameter were not in line with the predictions from the model, and even failed to replicate between the experiments. Experiment 1 revealed a significant effect of the content of thought, with a larger pupil size when participants had task-unrelated thoughts than during task-related thoughts. Although this result is in line with some previous studies (Franklin et al., 2013; Smallwood et al., 2012; Smallwood et al., 2011), it failed to replicate in Experiment 2, in which no significant effect on tonic pupillary diameter was found. The failure to find a clear effect of attentional state on the tonic pupillary diameter is not caused by oddities in the participants’ behavior or in our measurements. First, both our experiments replicated the typical results of the SART: Behavioral performance revealed faster RTs on trials prior to a no-go error than on trials prior to a correct no-go response (Cheyne et al., 2006; Allan Cheyne et al., 2009; Robertson et al., 1997), and a larger phasic pupillary response to the stimulus was observed for no-go than for go trials (Beatty & Lucero-Wagoner, 2000; Richer et al., 1983). Second, both experiments also replicated earlier findings in showing that the phasic pupillary response was smaller when participants were having thoughts that were unrelated to the task than when they were having task-related thoughts (Kang et al., 2014; Mittner et al., 2014; Smallwood et al., 2011; Unsworth & Robison, 2016, 2018).

It could also be argued that the inconsistent tonic pupil results were caused by an inadequate operationalization of the different attentional states proposed by Mittner et al. (2016). However, we think this is unlikely, because the relation between the attentional states and the RTCV matched the model predictions. Indeed, the behavioral results from both experiments revealed consistent findings that were in line with the model predictions. Behavioral variability captured by RTCV was found to be smallest during high-intensity task-related reports in both experiments, and it increased as intensity decreased. For task-unrelated reports, RTCV tended to show the opposite pattern, with a higher RTCV for high-intensity task-unrelated thoughts. Although this pattern failed to reach the significance threshold, the patterns were highly similar in both experiments and became significant when the data from both experiments were pooled. An intermediate RTCV was related to attentional states with low intensity reports, regardless of the content of thought (i.e., “off-focus” states). This not only replicated previous findings showing larger RTCVs when people were off task rather than on task (Bastian & Sackur, 2013), but also revealed the potential importance of the intensity dimension. RTCV only differed significantly when participants were having high-intensity thoughts; no significant difference was observed for on-task and MW states when the thoughts had a low intensity.

It should be noted that the intensity dimension and the different attentional states used in this study could resemble other dimensions or states that have been defined in MW research. For example, the “off-focus” state corresponds to a partial detachment from the current task with only a moderate impairment on behavioral performance (Mittner et al., 2016). According to this definition, the off-focus state could resemble what has been called “tuning out.” An active MW state is a deeper state of MW with a large impact on behavioral performance. As such, it is not unlike “zoning out.” However, research that has used tuning and zoning out (e.g., Dixon & Bortolussi, 2013; Smallwood et al., 2007) has related these states to awareness of the MW episodes (i.e., zoning out is typically related to unawareness of the episode, whereas tuning out is related to awareness of the episode). We therefore do not necessarily equate an off-focus and an active MW state to tuning out and zoning out, respectively, since awareness of the MW episode is not what distinguishes active MW from an off-focus state.

Our failure to find a reliable effect of intensity on tonic pupillary diameter means that the relation between tonic pupillary diameter and MW remains unclear. A recent study suggested that a difference in tonic activity could depend on the specific task instructions (Unsworth & Robison, 2018). Unsworth and Robison (2018) reported differences in tonic pupillary diameter between an on-task state and MW when the task promoted on-task behavior (e.g., when the ISI was variable or when the ISI was fixed but short), but not when the task promoted internal attention (e.g., when the ISI was fixed and long). It could be argued that the slow version of the SART used in our experiments promoted internal attention and that this is why no clear relation was found between tonic pupillary diameter and attentional states. Although this could indeed be the case—the reason why we chose the slow SART was exactly because it increases MW episodes—it should be noted that the ISI in our experiments was identical to the one that had shown a difference in tonic activity in the previous study (i.e., a fixed, short ISI of 2 s). Future research will need to establish the exact parameters that evoke differences in the tonic pupillary diameter for different attentional states.

An alternative explanation for the lack of a reliable relation between the tonic pupillary diameter and task-related and task-unrelated thoughts is that in our study—as in most other studies investigating this relation in humans—participants continued to perform the task even when they reported having task-unrelated thoughts or when their thoughts had the lowest intensity. This could suggest that participants were actively engaged in the task throughout the complete experiment, with their LC neurons tonically firing at a close-to-optimal level for the complete duration of the experiment. As a result, a relatively stable tonic pupillary diameter would be expected during the whole experiment. It is possible that only when participants’ minds seem to go nowhere or are completely empty (i.e., “mind blanking”; Ward & Wegner, 2013) can changes in LC firing be expected. Such a state in which a person experiences no thoughts and during which a stimulus fails to reach conscious awareness might be related to a more extreme version of our “off-focus” state. This mind-blanking state might be hard to achieve in tasks that require participants to continually react to a stimulus. In contrast, tasks without this requirement (e.g., reading a text) might be better at promoting mind-blanking, with differences in tonic pupillary diameter as a result (Franklin et al., 2013). The observed differences in phasic response to the stimuli could then be related to a higher optimization of the decision-related processes for task-related than for task-unrelated thoughts, which might be further tuned by the intensity of the thought. This difference in optimization between task-related and task-unrelated thoughts is also reflected in the observation that the RTCV was consequently smaller (i.e., less noisy) when participants were having task-related than when they were having task-unrelated thoughts.

Notwithstanding the absence of a reliable relation between MW and tonic pupillary diameter, a very strong relation between the pupil and MW was found for phasic pupillary response. Not only did this effect replicate between experiments in this study, it has also reliably been found in other studies (Mittner et al., 2014; Unsworth & Robison, 2016, 2018). Mittner et al. (2014), for example, also found a decrease in the phasic pupillary response to task-related stimuli while MW (Mittner et al., 2014). As with our results, they analyzed both tonic and phasic pupillary responses, and also suggested that the latter was the better predictor of attention state. Our results furthermore suggest that to predict the attentional state of a person according to objective criteria (i.e., without the need of a subjective report, as is the case in experience sampling), RTCV should be considered in combination with the phasic pupillary response. Research specifically tuning into the role of tonic pupil diameter in MW might want to try using other experimental tasks (such as reading tasks) that allow the participant’s mind to drift completely away from the task.

## Appendix: Instructions for thought probes

### Original instructions (in French)

*Pendant cette expérience, il vous sera demandé à différents moments (signalés par un “Bip”) si votre attention était totalement dirigée vers la tâche ou, inversement, si vous étiez concentré sur d’autres choses que la tâche uniquement. Par exemple, de façon occasionnelle, alors que vous regardez un chiffre, il peut arriver que vous commenciez à penser à des choses complètements non reliées à l’identification des chiffres. C’est ce qu’on appelle du “mind wandering” (= littéralement vagabondage de l’esprit) ou être “off task.”*

*Lorsqu’un signal retentit, vous devez faire attention au contenu de vos pensées: étiez-vous concentré sur la tâche? Ou étiez-vous concentré plutôt sur quelque chose d’autre (ex: qu’est ce que je vais manger ce soir?). En d’autres mots, avez-vous une idée claire du contenu de votre pensée? Ou, à l’inverse, alors que vous regardiez les chiffres, vous n’étiez concentré sur rien en particulier, ni sur la tâche ni sur autre chose.*

*En particulier, nous voulons que vous répondiez à ces questions:*

1. *Votre attention était-t-elle sur la tâche (on-task ) ou hors de la tâche (off-task)?*
2. *Sur une échelle de 1 à 4, comment qualifieriez-vous la netteté, la vivacité de ces pensées?*

*ex. off-task & 1: “Vous êtes assis dans un bus et vous réalisez que vous venez de rater votre arrêt alors que vous ne pensiez pas clairement à quelque chose d’autre.” Si on transfert cela à l’expérience: “je réalise la tâche sans y faire attention, de façon automatique, sans une idée claire de ce qui capte mon attention” (pas sur la tâche – faible netteté des pensées)*

*ex. on-task & 1: “Je réalise la tâche en faisant un peu attention, un peu de façon automatique, sans aucune autre idée qui capte mon attention” (sur la tâche – faible netteté des pensées)*

*ex. off-task & 4: “Faire la liste des articles je vais à acheter” (pas sur la tâche – forte netteté des pensées)*

*ex. on-task & 4: “Mon attention est clairement sur le chiffre” (sur la tâche – forte netteté des pensées)*

### Instructions (English translation)

During this experiment you will be asked at various points (marked by a “beep”) whether your attention is firmly directed towards the task, or alternatively you may be aware of other things than just the task. Occasionally you may find as you are staring at the numbers, that you begin thinking about something completely unrelated to identifying the numbers, as you press while other numbers appear; this is what we refer to as “mind wandering” or being “off-task.”

At the moment that the probe occurs, you have to pay attention to your thoughts: Were you focused on the task? or were you more focused on something else (e.g., what I will eat for dinner)? In other words, do you have a clear idea of the content of your thought? or on the other hand, while you were staring at the numbers, did not you have a clear focus on the task neither something else?

In particular, we want you to answer to these questions with two button presses:

1. Where was you attention? on-task vs. off-task (i.e., not on the task)
2. On a scale from 1 to 4, how would you classify the sharpness, the intensity, of your thought? 1 not clear focused thought – 4 very clear focused thought

e.g., off-task & 1: “You are sit inside a bus and you realize you have just missed your stop although you were not clearly thinking of something else.” In terms of the experiment: “I am performing the task without paying attention to it, in a kind of automatic way, with no clear thoughts taking my attention, if any.”

e.g., on-task & 1: “I am performing the task paying some but little attention to it, in a kind of automatic way, without other thoughts taking my attention.”

e.g., off-task & 4: “Listing up groceries that I need to buy.”

e.g., on-task & 4: “My attention is clearly focused on the numbers.”
